# On a New Efficient Steffensen-Like Iterative Class by Applying a Suitable Self-Accelerator Parameter

**DOI:** 10.1155/2014/769758

**Published:** 2014-03-03

**Authors:** Taher Lotfi, Elahe Tavakoli

**Affiliations:** Department of Mathematics, Hamedan Branch, Islamic Azad University, Hamedan 68138, Iran

## Abstract

It is attempted to present an efficient and free derivative class of Steffensen-like methods for solving nonlinear equations. To this end, firstly, we construct an optimal eighth-order three-step uniparameter without memory of iterative methods. Then the self-accelerator parameter is estimated using Newton's interpolation in such a way that it improves its convergence order from 8 to 12 without any extra function evaluation. Therefore, its efficiency index is increased from 8^1/4^ to 12^1/4^ which is the main feature of this class. To show applicability of the proposed methods, some numerical illustrations are presented.

## 1. Introduction

Kung and Traub are pioneers in constructing optimal general multistep methods without memory. They devised two general *n*-step methods based on interpolation. Moreover, they conjectured any *n*-step methods without memory using *n* + 1 function evaluations may reach the convergence order at most 2^*n*^ [1]. Accordingly, many authors during the last years, specially the four past years, are attempted to construct iterative methods without memory which support this conjecture with optimal order [1–22].

Although construction of optimal methods without memory is still an active field, however, much attention has not been paid for developing methods with memory. Based on our best knowledge, Traub in his book introduces the first method with memory. The main feature of these methods is that they improve convergence order as well as efficiency index without any new function evaluations. Indeed, Traub changed Steffensen's method slightly as follows (see [18, pp 185–187]):

(1) x0,w0,γ0  are  given  suitably,xn+1=xn−f(xn)f[xn,wn], 0≠γn∈R,  n=0,1,2,…,N1(x)=f(xn)+(x−xn)f[xn,wn],γn+1=−1N1′(xn),wn+1=xn+1+γn+1f(xn+1).

The parameter *γ*_*n*_ is called *self-accelerator* and method (1) has convergence order 2.41. It is still possible to increase the convergence order using better self-accelerator parameter based on better Newton interpolation. Free-derivative can be considered as another virtue of (1).

In this work, motivated by Traub's work (1), we construct a new class of methods with memory. To this end, we first try to devise a new optimal free-derivative three-step without memory of iterative methods with eight order of convergence and using merely four function evaluations per step. In other words, our first step is the same as Traub's method (1). The second and third steps use combination Steffensen-like methods and weight function idea so that we achieve an optimal class of methods without memory. Finally, we apply a self-accelerator parameter to extend it to with memory case. We remember two main properties of this work: increasing efficiency index without any new functional evaluations and nonusing derivatives of a given function.

We use the symbols →, *O*, and ~ according to the following conventions [18]: if lim⁡_*x*_*n*_→*∞*_ *g*(*x*_*n*_) = *C*, we write *g*(*x*_*n*_) → *C* or *g* → *C*. If lim⁡_*x*→*a*_ *g*(*x*) = *C*, we write *g*(*x*) → *C* or *g* → *C*. If *f*/*g* → *C*, where *C* is a nonzero constant, we write *f* = *O*(*g*) or *f* ~ *Cg*. Let *f*(*x*) be a function defined on an interval *I*, where *I* is the smallest interval containing *k* + 1 distinct nodes *x*_1_, *x*_2_,…, *x*_*k*_. The divided difference *f*[*x*_0_, *x*_1_,…, *x*_*k*_] with *k*th-order is defined as follows: *f*[*x*_0_] = *f*(*x*_0_)

(2) f[x0]=f[x1]−f[x0]x1−x0,…,f[x0,x1,…,xk]=f[x1,x2,…,xk]−f[x0,x1,…xk−1]xk−x0.

Moreover, we recall the definition of efficiency index (EI) as *E* = *p* ^1/*n*^, where *p* is the order of convergence and *n* is the total number of function evaluations per iteration.

This work is organized as follows: Section 2 present construction and error analysis of optimal three-step class of without memory class.  Section 3 is devoted to with memory extension. Numerical results are demonstrated in Section 5. We sum up this work in Section 5.

## 2. Derivative-Free Three-Point Method

This section concerns construction a new class of three-step free-derivative methods without memory for solving nonlinear equations. In the next section, it is extended to its with memory cases. To this end, let us first start with the following three-step Steffensen-type [23] initiative:

(3) yn=xn−f(xn)f[xn,wn],  wn=xn+γnf(xn),0≠γn∈R,  n=0,1,2,…,zn=yn−f(yn)f[yn,wn],xn+1=zn−f(zn)f[zn,wn].

This scheme is not optimal in the sense of Kung and Traub [1] as it is of fourth-order convergence using four functions evaluations per iteration. In other words, its error equation has the form

(4) $$\begin{array}{c} e_{n+1}={\left(1+f1a\gamma \right)}^{3}c_{2}^{3}e_{n}^{4}+O\left(e_{n}^{5}\right). \end{array}$$

Therefore, some modifications based on applying weight function ideas must be considered in such a way that the scheme (3) changes into an optimal method. Accordingly, we put forward the following iterative plan:

(5) $$\begin{array}{c} y_{n}=x_{n}-\frac{f\left(x_{n}\right)}{f\left[x_{n},w_{n}\right]},\\ z_{n}=y_{n}-H\left(t_{n},u_{n}\right)\frac{f\left(y_{n}\right)}{f\left[y_{n},w_{n}\right]},\\ x_{n+1}=z_{n}-G\left(t_{n},s_{n}\right)W\left(v_{n},s_{n}\right)\frac{f\left(z_{n}\right)}{f\left[z_{n},w_{n}\right]}, \end{array}$$

where *t*_*n*_ = *f*(*y*_*n*_)/*f*(*x*_*n*_), *u*_*n*_ = *f*(*w*_*n*_)/*f*(*x*_*n*_), *s*_*n*_ = *f*(*z*_*n*_)/*f*(*y*_*n*_), *v*_*n*_ = *f*(*z*_*n*_)/*f*(*x*_*n*_).

The main contribution of this section lies in the following Theorem which provides sufficient conditions for drawing optimal three-step iterations without memory class.

**Theorem 1**

Let *H*(*t*_*n*_, *u*_*n*_), *G*(*t*_*n*_, *s*_*n*_), and *W*(*v*_*n*_, *s*_*n*_) be differentiable two-variable functions that satisfy the conditions

(6) H(0,0)=H1,0(0,0)=1,H0,1(0,0)=H0,2(0,0)=H0,3(0,0)=H1,1(0,0)=H1,2(0,0)=H2,0(0,0)=H2,1(0,0)=0,G(0,0)=G1,0(0,0)=G0,1(0,0)=1,G2,0(0,0)=0,G1,1(0,0)=2,G3,0(0,0)=H3,0(0,0)−6−61+γnf[xn,wn],W(0,0)=1, W1,0(0,0)=W0,1(0,0)=0.

If the initial approximation *x*_0_ is sufficiently close to the zero *α* of a function *f*, then the convergence order of the family (5) is eight.

**Proof**

Let *e*_*n*_ = *x*_*n*_ − *α*, *e*_*y*_*n*__ = *y*_*n*_ − *α*, *e*_*z*_*n*__ = *z*_*n*_ − *α*, *e*_*w*_*n*__ = *e*_*n*_ + *γ*_*n*_ *f*(*x*_*n*_), and *c*_*n*_ = *f* ^(*n*)^  (*α*)/*n*!*f*′(*α*), *n* = 1,2,…. Using Taylor's expansion and taking into account *f*(*α*) = 0, we have

(7) f(xn)=f′(α)[en+c2en2+c3en3+c4en4+c5en5+c6en6+c7en7+O(en8)],f[xn,wn]=f′(α)[1+c2(2+γf′(α))en+(c22γf′(α)+c3(3+γf′(α)(3+γf′(α))))en2+⋯+O(en8)].

Substituting these into the first step of (5) gives

(8) eyn=en−f(xn)f[xn,wn]=c2(1+γf′(α))e2 +(c3(1+γf′(α))(2+γf′(α)) −c22(2+γf′(α)(2+γf′(α))))en3 +(c4(1+γf′(α))(3+γf′(α)(3+γf′(α))) +c23(4+γf′(α)(5+γf′(α)(3+γf′(α)))) −c2c3(7+γf′(α)(10+γf′(α)  ×(7+2γf′(α)))))en4 +⋯+O(en8).

Set *t*_*n*_ = *f*(*y*_*n*_)/*f*(*x*_*n*_), *u*_*n*_ = *f*(*w*_*n*_)/*f*(*x*_*n*_),  and *H*_*i*,*j*_ = ∂*H*(*t*_*n*_, *u*_*n*_)/∂*t*_*n*_^*i*^∂*u*_*n*_^*j*^, and expanding *H*(*t*_*n*_, *u*_*n*_) about (0,0), yields

(9) H(tn,un)=H0,0+H1,0tn+H0,1un +12(H2,0tn2+2H1,1tnun+H0,2un2) +⋯.

Substituting (9) into (5), we can assert that

(10) ezn=yn−H(tn,un)f(yn)f[yn,wn]=k1en2+k2en3+k4en4+k5en5+k6en6 +k7en7+O(en8),

where

(11) k1=−16(c2(1+γnf′(α)) ×(−6+6H0,0+3H0,2(1+γnf′(α))2+H0,3(1+γnf′(α))3 +6(H0,1+H0,1γnf′(α)))),

(12) k2=(−2c22+2c3−2c22γnf′(α)+3c3γnf′(α)−c22γn2f′(α)2+c3γn2f′(α)2−12c22(1+γnf′(α))×(2H1,1(γnf′(α))2+H1,2(γnf′(α))3+2(H1,0+H1,0γnf′(α))+γnf′(α)(2+γnf′(α))×(2H0,1+(1+γnf′(α))×(2H0,2+H0,3+H0,3γnf′(α))))+16(−c3(1+γnf′(α))(2+γnf′(α))+c22(3+2γnf′(α)(2+γnf′(α))))×(6H0,0+(1+γnf′(α))×(6H0,1+(1+γnf′(α))×(3H0,2+H0,3+H0,3γnf′(α)))))en3.

To achieve the fourth-order methods in the first two steps of (5), we attempt to vanish the coefficients of *e*_*n*_^2^, *e*_*n*_^3^ in (10). For this purpose, it suffices to set

(13) $$\begin{array}{c} H_{0,0}=H_{1,0}=1,\;H_{0,1}=H_{0,3}=H_{0,2}=H_{1,1}=H_{1,2}=0. \end{array}$$

Define *s*_*n*_ = *f*(*z*_*n*_)/*f*(*y*_*n*_),  *v*_*n*_ = *f*(*z*_*n*_)/*f*(*x*_*n*_), *G*_*i*,*j*_ = ∂*G*(*t*_*n*_, *s*_*n*_)/∂*t*_*n*_^*i*^∂*s*_*n*_^*j*^, and *W*_*i*,*j*_ = (∂*W*(*v*_*n*_, *s*_*n*_))/(∂*v*_*n*_^*i*^∂*s*_*n*_^*j*^), *i*, *j* = 1,2,…. Taylor's series for *G*(*t*_*n*_, *s*_*n*_), *W*(*v*_*n*_, *s*_*n*_) about (0,0) are

(14) G(tn,sn)=G0,0+G1,0tn+G0,1sn+12(G2,0tn2+2G1,1tnsn+G0,2sn2)+⋯,W(vn,sn)=W0,0+W1,0vn+W0,1sn+12(W2,0vn2+2W1,1vnsn+W0,2sn2)+⋯.

Under the conditions stated above (13) and substituting these Taylor's series into the third step of (5), we obtain

(15) en+1=zn−G(tn,sn)·W(vn,sn)f(zn)f[zn,wn]=R4en4+R5en5+R6en6+R7en7+O(en8),

where

(16) R4=12c2(−1+G0,0W0,0)(1+γnf′(α))2 ×(2c3+c22(H2,0+γnf′(α)H2,0+H2,1(1+γnf′(α))2−2(3+γnf′(α))).

Fix *G*_0,0_ = *W*_0,0_ = 1; then *R*_4_ = 0.

Assuming these conditions, (15) alters

(17) R5=12c22(−1+G1,0)(1+γnf′(α))3 ×(2c3+c22(H2,0+γnf′(α)H2,0+H2,1(1+γnf′(α))2−2(3+γnf′(α)))),

and to get *R*_5_ = 0, it is sufficient to put *G*_1,0_ = 1.

In the same manner, we can see that the coefficient of *e*_*n*_^6^ is

(18) R6=−14c2(1+γnf′(α))3 ×(2c3+c22(H2,0+γnf′(α)H2,0+H2,1×(1+γnf′(α)2−2(3+γnf′(α))))   ×(2c3(−1+G0,1+W0,1)   +c22(6+(−6+H2,0+H2,1)W0,1   +2γnf′(α)−G2,0(1+γnf′(α))))   +γnf′(α)W0,1  ×(−2+H2,0+H2,1(2+γnf′(α)))+G0,1  ×(H2,0+γnf′(α)H2,0+H2,1(1+γnf′(α))2  −2(3+γnf′(α)))).

To vanish the coefficient of *e*_*n*_^6^, set *G*_0,1_ = 1, *W*_0,1_ = *H*_2,0_ = *H*_2,1_ = *G*_2,0_ = 0, and we conclude similarly that

(19) R7=16c22(1+γnf′(α))4(−c3+c22(3+γnf′(α)))×(−6c3(−2+G1,1+W1,0)+c22(−24+18G1,1+G3,0−H3,0+18W1,0+γnf′(α)×(−6+6G1,1+G3,0−H3,0+6W1,0))).

As in the above cases, choosing *G*_1,1_ = 2, *W*_1,0_ = 0, and *G*_3,0_ = *H*_3,0_ − 6 − 6/(1 + *γ*_*n*_ *f*[*x*_*n*_, *w*_*n*_]) gives *R*_7_ = 0.

On account of the above conditions, we see that

(20) en+1=−16c2(1+γnf′(α))4 ×[(−c3+c22(3+γnf′(α)))×(−6c2c4+3c32(−2+G0,2+W0,2))−3c22c3(−22+6G0,2+G2,1+6W0,2+γnf′(α)×(−6+2G0,2+G2,1+2W0,2))+c24(−H3,0(1+γnf′(α))2+3G2,1(1+γnf′(α))(3+γnf′(α))+3G0,2(3+γnf′(α))2+3(W0,2(3+γnf′(α))2−2(13+γnf′(α)(7+γnf′(α)))))] ×en8+O[en9].

Some simple but efficient weight functions satisfying the conditions of Theorem 1 are

(21) H1(tn,un)=1+tn,H2(tn,un)=3+tntn+sin⁡(2tn(−2/9)tn2+3),G1(tn,sn)=1+tn+sn+2tnsn+(−1−ϕn)tn3,G2(tn,sn)=1/(1+ϕn)(1+tn+sn+2tnsn)+tn21/(1+ϕn)+tn2,

where *ϕ*_*n*_ = 1/(1 + *γ*_*n*_ *f*[*x*_*n*_, *w*_*n*_]).

Consider

(22) W1(sn,vn)=1+sn2+vn2,W2(sn,vn)=1+sn2vn2+1.

In the next section we introduce a new three-step method with memory. The efficiency index of the optimal class (5) is *E* = 8^1/4^. we extent proposed class (5) to its with memory version, using an accelerator parameter, which improves the efficiency index to 12^1/4^.

## 3. A New Method with Memory

Looking at the error equation (20) of the class (5) reveals that we can increase the convergence order of this class if the crucial element 1 + *γ*_*n*_ *f*′(*α*) vanishes. This can be done if *γ*_*n*_ = −1/*f*′(*α*). Although this is true theoretically, it is not possible practically since *α* is unknown. Fortunately, during the iterative process (5), finer approximations to *α* are generated by the sequence {*x*_*n*_}, and therefor we try to obtain a good approximate for *f*′(*α*). Each iteration, *x*_*n*_, *w*_*n*_,*y*_*n*_, *z*_*n*_, and *x*_*n*+1_, are accessible, except at the initial step. Hence, we can interpolate *f*′(*α*) using these nodes. It is natural that we estimate the best interpolator, and as a result we consider Newton interpolating polynomial as follows:

(23) N4′(xn)=[ddtN4(t;xn−1,wn−1,yn−1,zn−1,xn)]t=xn=[ddt(f(xn)+f[xn,zn−1](t−xn) +f[xn,zn−1,yn−1](t−xn)(t−zn−1) +f[xn,zn−1,yn−1,xn−1](t−xn) ×(t−zn−1)(t−yn−1) +f[xn,zn−1,yn−1,xn−1,wn−1](t−xn) ×(t−zn−1)(t−yn−1)(t−xn−1))]t=xn=f[xn,zn−1]+f[xn,zn−1,yn−1](xn−zn−1) +f[xn,zn−1,yn−1,xn−1](xn−zn−1)(xn−yn−1) +f[xn,zn−1,yn−1,xn−1,wn−1] ×(xn−zn−1)(xn−yn−1)(xn−xn−1).

In the next theorem we prove that if *γ*_*n*_≃−1/*N*_4_′(*x*_*n*_), then convergence order of the proposed class in Theorem 1 improves to 12.

**Theorem 2**

Suppose that *x*_0_ is an approximation to a simple zero *α* of *f*, then the *R*-order of convergence of the three-point method (5) is at least 12.

**Proof**

Suppose that an iterative method generates a sequence {*x*_*n*_} approximating a zero *α* of *f* and *C*_*n*_ tends to the asymptotic error constant *D*_*r*_ when *n* → *∞*, so

(24) $$\begin{array}{c} e_{n+1}\sim D_{n}e_{n}^{r},\;\;e_{n}=x_{n}-\alpha . \end{array}$$

Assume that the iterative sequences {*w*_*n*_}, {*y*_*n*_}, and {*z*_*n*_} have the *R*-order *p*, *q*, and *s*, respectively; that is,

(25) $$\begin{array}{c} e_{n,w}\sim A_{n}e_{n}^{p}=A_{n}{\left(D_{n-1}e_{n-1}^{r}\right)}^{p}=A_{n}D_{n-1}^{p}e_{n-1}^{rp},\\ e_{n,y}\sim B_{n}e_{n}^{q}=B_{n}{\left(D_{n-1}e_{n-1}^{r}\right)}^{q}=B_{n}D_{n-1}^{q}e_{n-1}^{rq},\\ e_{n,z}\sim C_{n}e_{n}^{s}=C_{n}{\left(D_{n-1}e_{n-1}^{r}\right)}^{s}=C_{n}D_{n-1}^{s}e_{n-1}^{rs},\\ e_{n+1}\sim D_{n}{\left(D_{n-1}e_{n-1}^{r}\right)}^{r}=D_{n}D_{n-1}^{r}e_{n-1}^{r^{2}}. \end{array}$$

On the other hand, based on error analysis of the Theorem 1, we have

(26) $$\begin{array}{c} e_{w_{n}}\sim \left(1+\gamma_{n}f^{\prime }\left(\alpha \right)\right)e_{n},\\ e_{y_{n}}\sim c_{2}\left(1+\gamma_{n}f^{\prime }\left(\alpha \right)\right)e_{n}^{2},\\ e_{z_{n}}\sim a_{k,4}{\left(1+\gamma_{n}f^{\prime }\left(\alpha \right)\right)}^{2}e_{n}^{4},\\ e_{n+1}\sim a_{k,8}{\left(1+\gamma_{n}f^{\prime }\left(\alpha \right)\right)}^{4}e_{n}^{8}, \end{array}$$

where *a*_*n*,4_ = −(1/2)*c*_2_(2*c*_3_ + *c*_2_^2^(*H*_2,0_(1 + *γ*_*n*_ *f*′(*α*)) + *H*_2,1_(1 + *γ*_*n*_ *f*′(*α*))^2^ − (3 + *γ*_*n*_ *f*′(*α*)))) and *a*_*n*,8_ are explicit from (20) and depend on iteration index since *γ*_*k*_ is recalculated in each step.

By (23) and the order of interpolatory iteration function, see Section 4.2 in [18], we can also conclude that

(27) $$\begin{array}{c} N_{4}^{\prime }\left(x_{n}\right)=f^{\prime }\left(\alpha \right)\left(1+c_{5}e_{n-1}e_{w_{n-1}}e_{y_{n-1}}e_{z_{n-1}}+\cdots \right). \end{array}$$

Since *γ*_*n*_ = −1/*N*_4_′(*x*_*n*_), then

(28) $$\begin{array}{c} 1+\gamma_{n}f^{\prime }\left(\alpha \right)\sim c_{5}e_{n-1}e_{w_{n-1}}e_{y_{n-1}}e_{z_{n-1}}. \end{array}$$

Combining (26) with (28), we infer that

(29) ewn~c5en−1ewn−1eyn−1ezn−1en~c5An−1Bn−1Cn−1Dn−1en−1r+p+q+s+1,eyn~c5en−1ewn−1eyn−1ezn−1en2~c2c5An−1Bn−1Cn−1Dn−12en−12r+p+q+s+1,ezn~an,4c52en−12ewn−12eyn−12ezn−12en4~an,4c52An−12Bn−12Cn−12Dn−14en−14r+2p+2q+2s+2,en+1~an,8c54en−14ewn−14eyn−14ezn−14en8~an,8c54An−14Bn−14Cn−14Dn−18en−18r+4p+4q+4s+4.

Equating powers on right-hand-side of relations (25) and (29), correspondingly, we form the following system of equations:

(30) $$\begin{array}{c} rq-r-s-p-q-1=0,\\ rp-2r-s-p-q-1=0,\\ rs-4r-2s-2p-2q-2=0,\\ r^{2}-8r-4s-4p-4q-4=0. \end{array}$$

Nontrivial solution of this system is *q* = 2, *p* = 3, *s* = 6, and *r* = 12. Therefore, the *R*-order of the methods with memory (5) under assumptions of Theorem 1, when *γ*_*n*_ = 1/*N*_4_′(*x*_*n*_), is at least 12.

**Remark 3**

If we use lower Newton interpolation, we achieve lower *R*-order.

## 4. Numerical Results

In this section, we test our proposed methods and compare their results with some other methods of the same order of convergence. First, we introduce some concrete methods based on the proposed class in this work.

Considering weight functions (21)–(22), we have

 Concrete method 1
  (31) x0,w0,γ0  are  given  suitably,yn=xn−f(xn)f[xn,wn], n=0,1,2,…,zn=yn−(1+tn)f(yn)f[yn,wn],xn+1=zn−(1+tn+sn+2tnsn+(−1−ϕn)tn3) ×(1+sn2+vn2)f(zn)f[zn,wn],γn+1=−1N4′(xn),wn+1=xn+1+γn+1f(xn+1).
 Concrete method 2
  (32) x0,w0,γ0  are  given  suitably,yn=xn−f(xn)f[xn,wn], n=0,1,2,…,zn=yn−(1+tn)·f(yn)f[yn,wn],xn+1=zn−(1/(1+ϕn)(1+tn+sn+2tnsn)+tn21/(1+ϕn)+tn2) ×(1+sn2vn2+1)f(zn)f[zn,wn],γn+1=−1N4′(xn),wn+1=xn+1+γn+1f(xn+1).
 Concrete method 3
  (33) x0,w0,γ0  are  given  suitably, n=0,1,2,…,yn=xn−f(xn)f[xn,wn],zn=yn−(3+tntn+sin2tn(−2/9)tn2+3)f(yn)f[yn,wn],xn+1=zn−(1/(1+ϕn)(1+tn+sn+2tnsn)+tn21/(1+ϕn)+tn2) ×(1+sn2+vn2)f(zn)f[zn,wn],γn+1=−1N4′(xn),wn+1=xn+1+γn+1f(xn+1).
 Concrete method 4
  (34) x0,w0,γ0  are  given  suitably,yn=xn−f(xn)f[xn,wn], n=0,1,2,…,zn=yn−(3+tntn+sin2tn(−2/9)tn2+3)f(yn)f[yn,wn],xn+1=zn−(1+tn+sn+2tnsn+(−1−ϕn)tn3) ×(1+sn2vn2+1)f(zn)f[zn,wn],γn+1=−1N4′(xn),wn+1=xn+1+γn+1f(xn+1).

For comparison purposes, we consider the following methods:

 Three-point by Sharma et al. [20]:
  (35) x0,w0,γ0  are  given  suitably,yn=xn−f(xn)f[wn,xn], n=0,1,2,…,zn=yn−1+un1−vnf(yn)f[wn,xn],xn+1=zn−f(zn) ×(f[zn,yn]+f[zn,yn,xn](zn−yn)+f[zn,yn,xn,wn](zn−yn)(zn−xn))−1,γn+1=−1N4′(xn),wn+1=xn+1+γn+1f(xn+1),
  where *u*_*n*_ = *f*(*y*_*n*_)/*f*(*x*_*n*_), and *v*_*n*_ = *f*(*y*_*n*_)/*f*(*w*_*n*_).
 Three-point by Kung and Traub [1]:
  (36) x0,w0,γ0  are  given  suitably,yn=xn−f(xn)f[wn,xn], n=0,1,2,…,zn=yn−f(yn)f(wn)[f(wn)−f(yn)]f[xn,yn], k=0,1,…,xn+1=zn−f(yn)f(wn)(yn−xn+f(xn)/f[xn,zn])[f(yn)−f(zn)][f(wn)−f(zn)] +f(yn)f[yn,zn],γn+1=−1N4′(xn),wn+1=xn+1+γn+1f(xn+1).
 Three-point by Zheng et al. [21]:
  (37) x0,w0,γ0  are  given  suitably,yn=xn−f(xn)f[xn,wn], n=0,1,2,…,zn=yn−f(yn)f[yn,wn]+f[yn,xn,wn](yn−xn),xn+1=zn−f(zn) ×(f[zn,yn]+f[zn,yn,xn](zn−yn)+f[zn,yn,xn,wn](zn−yn)(zn−xn))−1,γn+1=−1N4′(xn),wn+1=xn+1+γn+1f(xn+1).
 Three-point by Soleymani et al. [22]:
  (38) x0,w0,γ0  are  given  suitably,yn=xn−f(xn)f[wn,xn], n=0,1,2,…,zn=yn−f(yn) ×(f[yn,xn]+f[wn,xn,yn](yn−xn)+(yn−xn)(yn−wn))−1,xn+1=zn−f(zn) ×(f[xn,zn]+(f[wn,xn,yn]−f[wn,xn,zn]−f[yn,xn,zn])(xn−zn)+(zn−xn)(zn−wn)(zn−yn))−1,γn+1=−1N4′(xn),wn+1=xn+1+γn+1f(xn+1).

By |*x*_*n*_ − *α*| we denote approximations to the zero *α*, *b*(−*a*) stands for *b* × 10^−*a*^, and the computational order of convergence (COC). Here, COC is defined by [16]:

(39) $$\begin{array}{c} \text{COC}=\frac{\ln\left(\left|x_{n+1}-\alpha \right|/\left|x_{n}-\alpha \right|\right)}{\ln\left(\left|x_{n}-\alpha \right|/\left|x_{n-1}-\alpha \right|\right)}. \end{array}$$

Also the following functions are used:

(40) f(x)=exp⁡⁡(x2−3x)sin⁡(x)+log⁡⁡(x2+1), x0=0.35,  α=0,f(x)=exp⁡⁡(x2+xcos⁡⁡(x)−1)sin⁡(πx)+xlog⁡⁡(xsin⁡(x)+1), x0=0.6.

Tables 1 and 2 show numerical results for various optimal without memory methods (31)–(38). It is clear that all these methods behave very well practically and confirm their relevant theories.

Tables 3 and 4 present numerical results for various with memory methods (31)–(38). It is also clear that all these methods behave very well practically and confirm their relevant theories. They all provide 12th-order of convergence asymptotically without any new function evaluations.

## 5. Conclusions

In this work we proposed a new optimal class of methods without and with memory for computing simple root of a nonlinear equation. Its without and with memory methods attain 8 and 12 orders of convergence, respectively, using only four function evaluations per iterations. This class is free-derivative which can be considered as another virtue for it. All together, we managed to increase efficiency index of methods without memory from 8^1/4^ to 12^1/4^ using a very suitable self-accelerator parameter based on Newton interpolation.

## Figures and Tables

**Table 1:** *f*(*x*) = exp⁡(*x* ^2^ − 3*x*)sin⁡(*x*) + log⁡(*x* ^2^ + 1), *x*_0_ = 0.35, α = 0, γ = 1.

Methods	|*x*_1_ − α|	|*x*_2_ − α|	|*x*_3_ − α|	COC (39)
New Method (31)	0.61569 (−3)	0.23067 (−21)	0.91264 (−169)	7.999
New Method (32)	0.56124 (−3)	0.32323 (−21)	0.38634 (−167)	8.000
New Method (33)	0.55232 (−3)	0.28429 (−21)	0.13833 (−167)	8.000
New Method (34)	0.62453 (−3)	0.25853 (−21)	0.22480 (−168)	7.999
Method (35)	0.19676 (−4)	0.44197 (−33)	0.28657 (−262)	8.000
Method (36)	0.85597 (−4)	0.28686 (−29)	0.45644 (−233)	8.000
Method (37)	0.17236 (−4)	0.32121 (−35)	0.46744 (−281)	8.000
Method (38)	0.34824 (−4)	0.36172 (−33)	0.48972 (−265)	8.000

**Table 2:** *f*(*x*) = exp⁡(*x* ^2^ + *x*cos⁡(*x*) − 1)sin⁡(π*x*) + *x*log⁡(*x*sin⁡(*x*) + 1), *x*_0_ = 0.6, α = 0, γ = −1.

Methods	|*x*_1_ − α|	|*x*_2_ − α|	|*x*_3_ − α|	COC (39)
New Method (31)	0.57578 (−3)	0.71057 (−29)	0.38797 (−236)	7.999
New Method (32)	0.45807 (−3)	0.5295 (−31)	0.35165 (−254)	7.999
New Method (33)	0.46574 (−3)	0.65400 (−31)	0.10135 (−253)	7.999
New Method (34)	0.56806 (−3)	0.63795 (−29)	0.16377 (−236)	7.999
Method (35)	0.48202 (−3)	0.27805 (−30)	0.34404 (−248)	7.999
Method (36)	0.31009 (−3)	0.26712 (−31)	0.841965 (−256)	7.999
Method (37)	0.23448 (−3)	0.10417 (−32)	0.15929 (−267)	7.999
Method (38)	0.46334 (−3)	0.23577 (−30)	0.10713 (−248)	7.999

**Table 3:** *f*(*x*) = exp⁡(*x* ^2^ − 3*x*)sin⁡(*x*) + log⁡(*x* ^2^ + 1), *x*_0_ = 0.35, α = 0, γ_0_ = 0.01.

Methods	|*x*_1_ − α|	|*x*_2_ − α|	|*x*_3_ − α|	COC (39)
New Method (31)	0.91937 (−4)	0.18790 (−44)	0.11705 (−532)	11.998
New Method (32)	0.11053 (−3)	0.77050 (−44)	0.31600 (−525)	11.988
New Method (33)	0.11306 (−3)	0.99434 (−44)	0.67431 (−524)	11.987
New Method (34)	0.89413 (−4)	0.13787 (−44)	0.28524 (−534)	11.998
Method (35)	0.14850 (−5)	0.17577 (−61)	0.48167 (−738)	12.097
Method (36)	0.84533 (−4)	0.39381 (−45)	0.10032 (−540)	11.991
Method (37)	0.30874 (−6)	0.17978 (−67)	0.12617 (−812)	12.169
Method (38)	0.16768 (−4)	0.15361 (−56)	0.21643 (−680)	11.988

**Table 4:** *f*(*x*) = exp⁡(*x* ^2^ + *x*cos⁡(*x*) − 1)sin⁡(π*x*) + *x*log⁡(*x*sin⁡(*x*) + 1), *x*_0_ = 0.6, α = 0, γ_0_ = −0.1.

Methods	|*x*_1_ − α|	|*x*_2_ − α|	|*x*_3_ − α|	COC (39)
New Method (31)	0.71066 (−4)	0.20396 (−49)	0.49715 (−596)	12.002
New Method (32)	0.80715 (−4)	0.15495 (−49)	0.65738 (−595)	11.929
New Method (33)	0.78950 (−4)	0.14520 (−49)	0.30139 (−596)	11.931
New Method (34)	0.72833 (−4)	0.22472 (−49)	0.15905 (−595)	12.000
Method (35)	0.64946 (−4)	0.48258 (−50)	0.11725 (−600)	11.936
Method (36)	0.60478 (−4)	0.17480 (−48)	0.27838 (−582)	11.985
Method (37)	0.65693 (−4)	0.27775 (−50)	0.42521 (−607)	11.993
Method (38)	0.86612 (−4)	0.81112 (−64)	0.23877 (−765)	12.089

## References

[1] Kung HT, Traub JF (1974). Optimal order of one-point and multipoint iteration. *Journal of the Association for Computing Machinery*.

[2] Behl R, Kanwar V, Sharma KK (2012). Another simple way of deriving several iterative functions to solve nonlinear equations. *Journal of Applied Mathematics*.

[3] Soleimani F, Soleymani F, Shateyi S (2013). Some iterative methods free from derivatives and their basins of attraction for nonlinear equations. *Discrete Dynamics in Nature and Society*.

[4] Fernandez-Torres G, Vásquez-Aquino J (2013). Three new optimal fourth-order iterative methods to solve nonlinear equations. *Advances in Numerical Analysis*.

[5] Montazeri H, Soleymani F, Shateyi S, Motsa SS (2012). On a new method for computing the numerical solution of systems of nonlinear equations. *Journal of Applied Mathematics*.

[6] Soleymani F (2011). Novel computational iterative methods with optimal order for nonlinear equations. *Advances in Numerical Analysis*.

[7] Soleymani F, Karimi Vanani S, Afghani A (2011). A general three-step class of optimal iterations for nonlinear equations. *Mathematical Problems in Engineering*.

[8] Soleymani F, Sharifi M, Mousavi S (2012). An improvement of Ostrowski’s and King’s techniques with optimal convergence order eight. *Journal of Optimization Theory and Applications*.

[9] Soleymani F, Karimi Vanani S, Jamali Paghaleh M (2012). A class of three-step derivative-free root solvers with optimal convergence order. *Journal of Applied Mathematics*.

[10] Soleymani F, Karimi Vanani S, Khan M, Sharifi M (2012). Some modifications of King’s family with optimal eighth order of convergence. *Mathematical and Computer Modelling*.

[11] Thukral R (2012). Further development of Jarratt method for solving nonlinear equations. *Advances in Numerical Analysis*.

[12] Thukral R (2011). Eighth-order iterative methods without derivatives for solving nonlinear equation. *ISRN Applied Mathematics*.

[13] Thukral R (2012). New eighth-order derivative-free methods for solving nonlinear equations. *International Journal of Mathematics and Mathematical Sciences*.

[14] Thukral R (2010). A new eighth-order iterative method for solving nonlinear equations. *Applied Mathematics and Computation*.

[15] Kang SM, Rafiq A, Kwun YC (2013). A new second-order iteration method for solving nonlinear equations. *Abstract and Applied Analysis*.

[16] Soleymani F (2013). A new method for solving ill-conditioned linear systems. *Opuscula Mathematica*.

[17] Soleymani F (2012). A rapid numerical algorithm to compute matrix inversion. *International Journal of Mathematics and Mathematical Sciences*.

[18] Traub JF (1964). *Iterative Methods for the Solution of Equations*.

[19] Wang J (2013). He’s max-min approach for coupled cubic nonlinear equations arising in packaging system. *Mathematical Problems in Engineering*.

[20] Sharma JR, Guha RK, Gupta P (2012). Some effcient derivative free methods with memory for solving nonlinear equations. *Applied Mathematics and Computation*.

[21] Zheng Q, Li J, Huang F (2011). An optimal Steffensen-type family for solving nonlinear equations. *Applied Mathematics and Computation*.

[22] Soleymani F, Babajee DKR, Shateyi S, Motsa SS (2012). Construction of optimal derivative-free techniques without memory. *Journal of Applied Mathematics*.

[23] Steffensen JF (1933). Remarks on iteration. *Skand Aktuar Tidsr*.

